# Occurrence and Variability of the Efflux Pump Gene *norA* across the *Staphylococcus* Genus †

**DOI:** 10.3390/ijms232315306

**Published:** 2022-12-04

**Authors:** Carolina Ferreira, Patrícia Abrantes, Sofia Santos Costa, Miguel Viveiros, Isabel Couto

**Affiliations:** Global Health and Tropical Medicine, GHTM, Instituto de Higiene e Medicina Tropical, IHMT, Universidade NOVA de Lisboa, UNL, 1349-008 Lisbon, Portugal

**Keywords:** *Staphylococcus aureus*, *Staphylococcus*, *Mammalliicoccus*, *norA*, allele, efflux pump, genetic diversity, phylogenetic analysis, screening, core genome

## Abstract

NorA is one of the main native MDR efflux pumps of *Staphylococcus aureus*, contributing to reduced susceptibility towards fluoroquinolones and biocides, but little is known about its variability within *S. aureus* or its distribution and conservation among other staphylococci. We screened for sequences homologous to *S. aureus norA* and found it in 61 out of the 63 *Staphylococcus* species described. To the best of our knowledge, this is the first study to report the occurrence of *norA* across the *Staphylococcus* genus. The *norA* phylogenetic tree follows the evolutionary relations of staphylococci and the closely related *Mammalliicoccus* genus. Comparative analyses suggest a conservation of the NorA function in staphylococci. We also analyzed the variability of *norA* within *S. aureus*, for which there are several circulating *norA* alleles, differing up to 10% at the nucleotide level, which may hamper proper *norA* detection. We demonstrate the applicability of a PCR-based algorithm to detect and differentiate *norA* alleles in 52 *S. aureus* representing a wider collection of 89 isolates from different hosts. Our results highlight the prevalence of *norAI* and *norAII* in different settings and the association of *norA* alleles with specific *S. aureus* clonal lineages. Ultimately, it confirms the applicability of our PCR-based algorithm to rapidly detect and assign the different *norA* alleles, a trait that may impact antimicrobial efflux capacity and the search for potential NorA inhibitors.

## 1. Introduction

NorA was the first multidrug efflux pump (MDR EP) to be described in *Staphylococcus aureus* [1] and remains the most well-studied MDR EP of the several native efflux systems described for this bacterium. NorA is a transporter of the major facilitator superfamily (MFS) and is composed of 12 transmembrane segments (TMS) and 388 amino acids [2,3]. Early reports associated this MDR EP with extrusion of a wide range of antimicrobial agents that include fluoroquinolones (particularly with hydrophobic character), several biocides such as quaternary ammonium compounds (cetrimide, benzalkonium chloride), and dyes (ethidium bromide, rhodamine, acriflavine) [1,4]. In the last decade, additional substrates of NorA have been identified, namely siderophores [5] and fusaric acid [6]. The contribution of NorA to the reduced susceptibility towards fluoroquinolones and biocides in *S. aureus* isolates of human or environmental origin has been demonstrated by several studies [7,8,9,10,11], as well as its role as a first-step resistance mechanism towards these antimicrobial agents [12,13]. Besides *S. aureus*, less is known about the genetic occurrence of *norA* among other staphylococci. There is scarce literature on the NorA of *S epidermidis*, *S. pseudintermedius*, and *S. haemolyticus* and its contribution to fluoroquinolone resistance and/or reduced susceptibility to biocides [14,15,16], and little is known about the presence of this MDR EP across the entire *Staphylococcus* genus.

NorA is encoded by the 1164 bp *norA* gene. The genetic variability of *S. aureus norA* was reported earlier in literature with three *norA* alleles described, namely *norAI* [2], *norAII* [17], and *norAIII* [18]. These gene variants differed by up to 10% and 5% at the nucleotide and polypeptide sequence, respectively. Failure to recognize the inherent variability of the NorA coding gene still results in many reports in the literature describing *S. aureus* strains as not bearing *norA* due to failure to conduct amplification with the appropriate set of primers. Another confounding factor is the lack of recognition of *norA* as an *S. aureus* core gene with inherent variability in repositories that feed bioinformatic tools for analysis of antibiotic-resistance genes.

In a previous work from our group, we demonstrated that *norA* is a core gene of *S. aureus* and that each strain carries one of the several *norA* alleles [19]. We also reported that besides the three *norA* alleles already described in the literature, there was at least a fourth allele and potentially several other variants. Additionally, we observed a relation between the different *norA* alleles and specific *S. aureus* lineages [19]. Among the databases analyzed, we observed a prevalence of *norAI* and *norAII* alleles, since these are related to the current predominant *S. aureus* clonal complexes, including CC5, CC8, and CC22. In that earlier study, we proposed a molecular approach for the rapid recognition of the different circulating *norA* alleles based on a set of primers designed to differentiate the four known *norA* alleles [19]. 

We now demonstrate the applicability of this molecular approach to detect *norA* variability among a set of contemporary *S. aureus* isolates of both human and companion animal origin, further demonstrating the occurrence of different *S. aureus norA* alleles and their relationship with specific *S. aureus* clonal lineages. We also extend our analysis to the remaining *Staphylococcus* genus by screening the presence of a *norA* determinant in the genomes of strains representative of 61 staphylococcal species and the closely related *Mammalliicoccus* genus and by proposing that this element is part of the staphylococcal core genome.

## 2. Results and Discussion

### 2.1. The norA Gene Is Ubiquitous across the Staphylococcus Genus

To better understand if *norA* is ubiquitous within the *Staphylococcus* genus, sequences homologous to *S. aureus norA* were screened in available databases. The retrieved nucleotide sequences of the *norA* gene from different *Staphylococcus* species were aligned and studied in terms of phylogeny. The corresponding polypeptide sequences were analyzed, and the impact of possible residue substitutions on NorA activity was predicted.

A total of 61 nucleotide sequences representing the *norA* gene from 61 out of the 63 staphylococcal species described to date was found in the available genome databases (Supplementary Material S1), highlighting that the corresponding efflux pump is part of the fundamental machinery of the staphylococcal cell. The two species not included in this analysis were *S. canis*, for which we only found an incomplete *norA* sequence, and *S. massiliensis,* for which no *norA* related sequence was found, possibly because its genome is not yet fully sequenced. 

A multiple alignment was performed for the 61 nucleotide sequences retrieved together with the three *S. aureus norA* prototype alleles. The *norA* sequences of the five species that were recently reclassified to the new genus *Mammalliicoccus*, namely *M. sciuri* (formerly *S. sciuri*), *M. fleurettii* (formerly *S. fleurettii*), *M. vitulinus* (formerly *S. vitulinus*), *M. lentus* (formerly *S. lentus*), and *M. stepanovicii* (formerly *S. stepanovicii*) were also included in the alignment due to the historical and phylogenetic relatedness with the *Staphylococcus* genus. The highest *norA* identity among the species analyzed in this study was between *M. fleurettii* and *S. schleiferi*, with 100.0% sequence identity (which may reflect *M. fleurettii* misassignment in databases), while the largest nucleotide difference was found between *S. delphini* and *M. vitulinus* and corresponded to 54.5% identity

**Figure 1:**
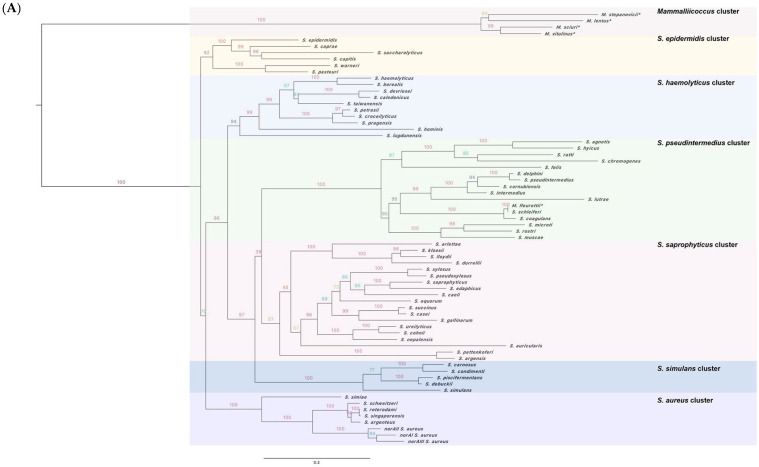

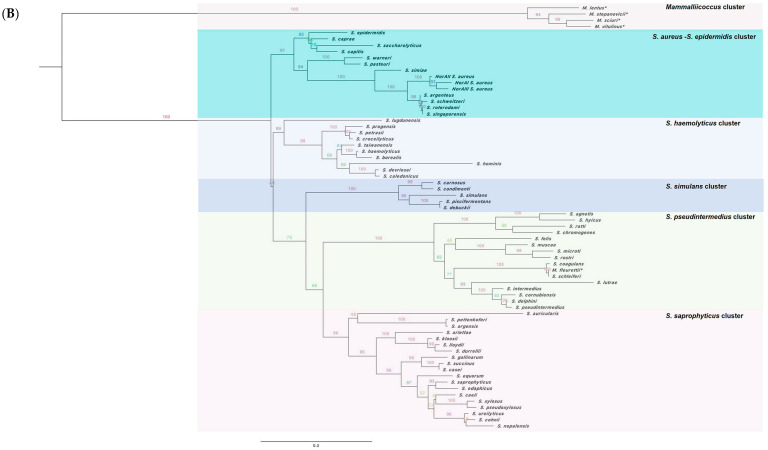
Phylogenetic analysis of the *norA* efflux pump gene (**A**) and NorA efflux pump (**B**) across the *Staphylococcus* genus. Maximum likelihood (ML) consensus tree rooted at midpoint and drawn to scale, with branch lengths in the scale of nucleotide (**A**) or aminoacid (**B**) substitutions per site. Bootstrap support values are illustrated at branch nodes. The species highlighted by * correspond to the species that were recently proposed to be reclassified from *Staphylococcus* to the new *Mammalliicoccus* genus.

As shown in Figure 1A, the *norA* phylogenetic tree reconstructed by the maximum likelihood method identified seven cluster groups. A monophyletic group containing *M. lentus*, *M. sciuri*, *M. vitulinus* and *M. stepanovicii* was identified as the sister group to all other *Staphylococcus* analyzed, a result in line with the recent assignment of these four species to the new *Mammalliicoccus* genus [20]. As expected, *norAI*, *norAII,* and *norAIII* from *S. aureus* formed a strongly supported sub-cluster (bootstrap = 100%), together with *S. argentus*, *S. schweitzeri*, *S. roterodami*, and *S. singaporensis*—four recently renamed species that were previously described as divergent lineages of *S. aureus* [21,22,23]. Except for *M. fleurettii*, also recently reassigned to the new *Mammalliicoccus* genus, most relations within the *norA* gene phylogenetic tree are in general agreement with the evolutionary relations of staphylococci based on the phylogenetic tree of 16S-23S rRNA region obtained by Kosecka-Strojel et al. (2019) [24], highlighting the presence of *norA* in the early branching of the *Staphylococcus* genus.

### 2.2. Characterization of the NorA Predicted Polypeptide Sequences across Staphylococci

Figure 1B illustrates the phylogenetic tree based on NorA polypeptide sequences built using a maximum likelihood tree, showing a similar evolutionary relation at the nucleotide and at the protein level. An exception was observed for *S. epidermidis* and *S. aureus* clusters that are merged at the polypeptide level.

**Figure 2:**
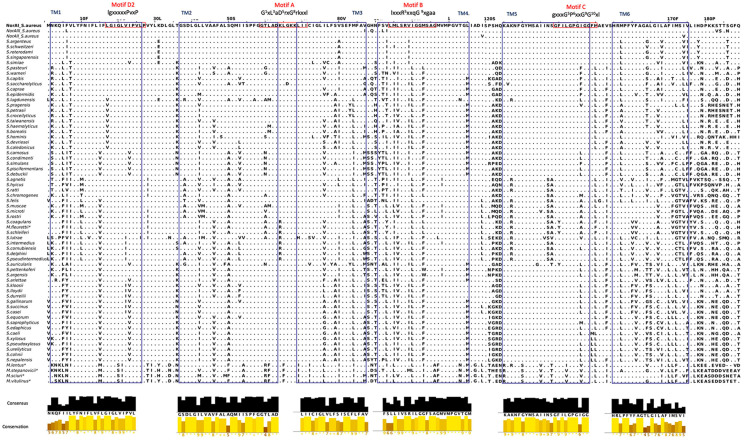

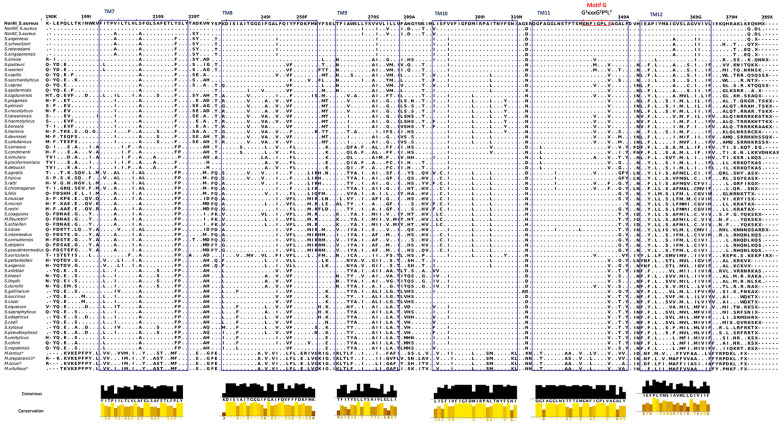
Multiple alignment of NorA polypeptide sequences derived from *S. aureus* alleles *norAI* [2], *norAII* [17], and *norAIII* [18] and from the *norA* sequences identified for other *Staphylococcus* species. Blue boxes correspond to the transmembrane segments (TMS) of NorA predicted by Brawley et al. [28] and red boxes to the MFS conserved motifs predicted by Paulsen et al. [25]. Consensus and conservation tracks are displayed for each of the TMS identified. Consensus histogram (black) reflects the percentage of the modal residue per column (‘+’ denotes non-conserved residues). Conservation histogram (yellow) reflects conservation of the physicochemical properties (‘*’ absolutely conserved residues, ‘+’ physicochemical properties conserved; less conserved positions are shown in darker colors with decreasing score <9).

Figure 2 displays the alignment of the 68 polypeptide sequences of NorA and NorA-like found across staphylococci and the newly established *Mammalliicoccus* genus. Identity of NorA sequences among *Staphylococcus* species varies between 64.0% and 100.0%. The highest divergency was observed between *S. simulans* and *S. lutrae* (140 residues difference), while the most closely related protein sequences were observed between *M. fleuretti* and *S. schleiferi* and between *S. roterodami* and *S. singaporensis*, corresponding to 100% identity.

Figure 2 summarizes these alterations and their location within the predicted NorA sequences as well as consensus and conservation histograms for each TMS when compared to NorAI of *S. aureus*. This comparative analysis indicates a higher divergence on the C-terminal region, in agreement with the knowledge that efflux pumps from the MFS 12-TMS families share greater sequence similarity within their N-termini [25]. As observed for other MFS pumps, the predicted NorA presents high percentage of glycines in TMS5 and an overall conservation of this TMS among the different staphylococcal species. Abundance of glycines in TMS5 has been postulated to confer conformational plasticity to efflux pumps [26].

We also observed a high divergence in the polypeptide region between the putative transmembrane segments TMS6 and TMS7 (residues 176–202), within TMS9 and TMS12 and in C-terminal, while the least divergent region is located between residues 300–345, encompassing the transmembrane segments TMS10 and TMS11. Several residue substitutions were found within MFS conserved motifs [25,27]. Motif A (G^1^xL^3^aD^5^rxG^8^rkxxl), which includes the cytoplasmatic loop between TMS2 and TMS3, is mostly conserved, yet residue substitutions at the position L^3^ are encountered in a few species of the *S. haemolyticus* cluster (Leu61Met) and in the *Mammalliicoccus* cluster (Leu61Phe). Motif B (lxxxR^5^xxqG^9^xgaa), located within TMS4; Motif C (gxxxG^5^P^6^xxG^9^G^10^xl), located within TMS5; and Motif G (G^1^xxxG^5^P^6^L^7^) within TMS11 were mostly conserved throughout all predicted NorA sequences.

A recent study by Brawley and colleagues determined the first *S. aureus* NorA structure, complexed with a synthetic antigen-binding fragment (Fab36) [28]. This study revealed eleven residues that may play an important functional role in NorA, since their substitution significantly affected resistance to norfloxacin, a substrate of NorA. We observed a conservation of six out of those eleven residues through *Staphylococcus* spp. and that the residue substitutions were mostly specific to some of the NorA clusters (Figure 1B and Figure 2). The alterations Gly20Ser and Thr336Ala were only detected in the *Mammalliicoccus* cluster, whereas the alterations Asn137Ser and Phe140Tyr were found in all or some of the species included in the *S. pseudintermedius* cluster, respectively. Additionally, the Ile23Val substitution was detected in the *S. simulans* cluster and all species of the *S. saprophyticus* cluster except for *S. pettenkoferi* and *S. argensis*. We expanded this analysis using the SuSPect algorithm with the NorA structural model described by Brawley and colleagues [28] to predict deleterious variants in the protein. The differences encountered between the several NorA polypeptide sequences correspond to residues that were not predicted to be pivotal for the protein activity (Supplementary Material S2), again reinforcing a conservation of NorA function across staphylococci.

In another recent study, Shang and colleagues describe alterations in the 277–297 polypeptide region that have a significant impact on NorA efflux activity and resistance to fluoroquinolones and could be partially responsible for the functional differences of the NorA EP in *S. aureus*, particularly the substitutions Val281Ile, Phe288Ile, and Asn290Asp, suggesting that the 277–297 region plays a major role in NorA conformational stabilization [29]. However, this region is significantly variable among staphylococcal NorA sequences analyzed in our study. In particular, residues Val281, Phe288, and Asn290 present several different alterations across staphylococci, including in species for which NorA activity and its association with fluoroquinolone resistance has already been demonstrated, namely *S. epidermidis* [14] and *S. pseudintermedius* [16], suggesting that additional residues may be involved in the conformational stabilization of the NorA family.

### 2.3. Applicability of a Molecular Approach for Rapid norA Allele Screening in S. aureus

After establishing the presence of the *norA* determinant among the entire *Staphylococcus* genus, we then focused our study on the variability of this determinant within *S. aureus*. We have previously established that *norA* is a core gene of *S. aureus* and that each strain carries one of several *norA* alleles. This led to the proposal of a molecular approach for the rapid detection of the different *norA* alleles as failure to detect a *norA* variant may result into misleading interpretations [19].

The primers proposed earlier to screen this allelic variability [19] were now applied as suggested, with few modifications (Table 1), to our study collection of 52 *S. aureus* strains of human (*n* = 25) or companion animal (*n* = 27) origin, representative of all the PFGE types or sub-types previously detected in a wider collection of 89 *S. aureus* strains. The main characteristics of these strains have been described elsewhere, including antibiotic susceptibility and molecular typing [30,31]. Briefly, the 25 strains of human origin comprised 16 clonal lineages, as defined by MLST, corresponding mainly to clonal complexes CC5, CC7, CC8, CC15, CC22, CC25, CC30, CC45, CC97, CC152 [30]. The 27 *S. aureus* from veterinary sources included 14 clonal lineages belonging to CC1, CC5, CC7, CC8, CC15, CC22, CC97, CC121, and CC398 [31].

A *norA* amplification product was obtained for each strain tested, further confirming our earlier observation that the *norA* gene is part of the *S. aureus* core genome. Additionally, a single amplification product was obtained with the three sets of primers used for 50 out of the 52 strains tested (Table 2). The only exceptions were the two *S. aureus* carrying the *norA_CC59_*–*_CC121_* allele that amplified with the primers for *norAIII*/*norA_CC59_*–*_CC121_* and with the ones for *norAI* (Table 2).

Our previous study suggested that each *norA* allele is related to specific *S. aureus* clonal lineages [19], which was confirmed in the present work. For example, *S. aureus* belonging to CC5 and CC8, predicted to carry the *norAI* allele, only produced an amplification product when subjected to the *norAI*-specific PCR, whereas those belonging to CC22 and CC398, predicted to harbor *norAII* allele, were only positive in the *norAII*-specific PCR (Table 2). The association of *norAIII* allele and CC45 was also confirmed, considering that the single ST278 strain belongs to the CC45 clonal lineage. On the other hand, the strain belonging to ST121, expected to carry *norA_CC59_*–*_CC121_*, showed an unexpected result by producing amplicons with both *norAI* and *norAIII*/*norA_CC59_*–*_CC121_* primers. Nevertheless, when subjected to enzymatic digestion with *Hind*III, the *norAIII*/*norA_CC59_*–*_CC121_*-PCR amplicon was not digested, as expected for a *norA_CC59_*–*_CC121_* allele (Table 2).

Some of the strains tested belong to clonal lineages for which no association with a particular *norA* allele has been established yet, namely CC7, CC25, CC97, and the singletons ST816 and ST6564. By applying our molecular approach, we were able to assign a *norA* allele to each one of these strains (Table 2). In particular, *norAI* was found in strains from lineages CC7, CC25, and CC97, while *norAII* was associated with ST6564. The ST816 strain presented a pattern similar to the one obtained for the ST121 strain, indicating that ST816 strain also harbors *norA_CC59_*–*_CC121_* (Table 2).

**Figure 3:**
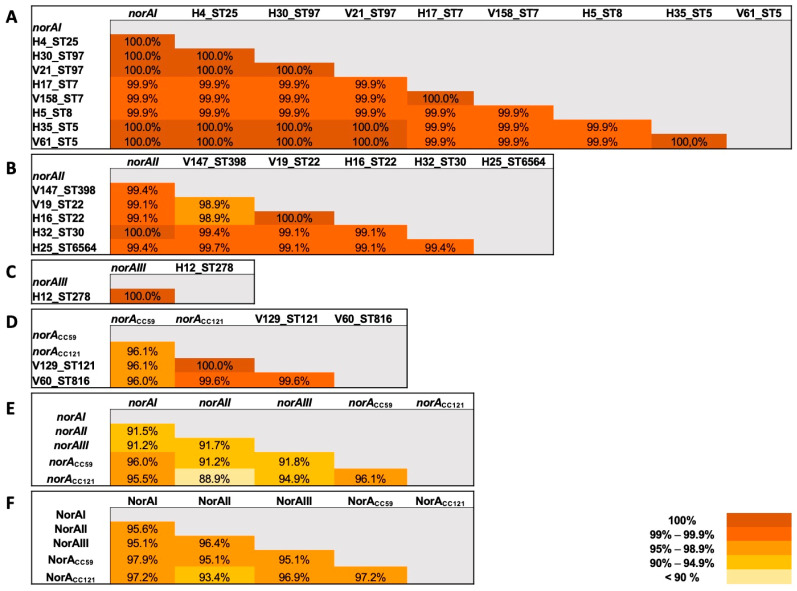
Variability between and within *norA* alleles (**A***–***E**) and between NorA proteins (**F**) for the *S. aureus* strains studied in comparison with prototype alleles and their products. Comparisons were made with MEGA 7.0.26 software and are indicated as the identity between sequences (%). A color code highlights the degree of identity between the compared *norA*/NorA sequences.

To further confirm the assignment of *norA* allelic profiles, we sequenced the entire *norA* gene in a subset of 16 strains belonging to the 12 main clonal lineages present in the collection. For each strain, we amplified, sequenced, and assembled three PCR products to obtain the full 1164 bp sequence of *norA*. For strains belonging to ST22, ST5, ST7, and ST97, *norA* sequences were obtained for two strains, either from human or companion animal origin. Multiple alignment of the 16 *norA* sequences with each prototype allele confirmed the correct PCR-based allelic assignment. As expected, each strain tested harbored a *norA* allele with up to 10 nucleotides of difference toward the respective prototype *norA*. Of them, *norAI* showed less variability, with nucleotide identity ranging from 99.9% (a single nucleotide variation) to 100% (Figure 3A). The *norAII* allele was more variable, with nucleotide identities varying from 98.9% to 100% (Figure 3B). The single *norAIII* detected showed no variation to the prototype allele (Figure 3C). Regarding the *norA_CC59_*–*_CC121_* allele, the analysis was conducted against the *norA* sequences retrieved from two strains of clonal lineages ST59 and ST121 with complete genomes available at GenBank Database. Nucleotide identities varied from 99.6% to 100% when aligned with the ST121-associated *norA* and from 96.0% to 96.1% when aligned with *norA* from the ST59 strain (Figure 3D).

Although limited, the variations observed among the different *norA* alleles may impact NorA efflux activity and therefore susceptibility towards several antimicrobial agents. Considering the intra-allelic variation, most of the alterations found were silent and only a few resulted in alterations in the polypeptide sequence. Regarding the *norAI* allele, we found a nucleotide variation in one ST8 strain that resulted in the substitution Gly291Asp. This same alteration had already been identified in previous studies, both in fluoroquinolone-susceptible and -resistant strains, thus it is not expected to affect NorA activity [33]. Regarding the *norAII* allele, we found the substitution Asn200Asp in two ST22 strains. It was not possible to ascertain the intra-allelic variation of *norAIII*, since this less frequent allele was only detected in a single strain. Regarding the *norA_CC59_*–*_CC121_* allele, there are several nucleotide alterations between the *norA* CC59 and *norA* CC121 prototype sequences that yield 11 amino acid variations. The strain belonging to ST816 has three and 10 amino acid alterations when compared to the CC121 and CC59 sequences, respectively (Figure 3D).

In terms of inter-allelic variability, the *norAI* allele has ~91% identity at the nucleotide level with the *norAII* and *norAIII* alleles and ~96% identity with the *norA_CC59_*–*_CC121_* allele (Figure 3E). The *norAII* allele has ~91% identity with the *norAIII* and the *norA_CC59_*–*_CC121_* allele, but the identity is lower (88.9%) in comparison with the CC121 reference *norA*. The *norAIII* allele has 91.8% to 94.9% identity with the *norA_CC59_*–*_CC121_* allele. An identity of 96.1% was also found between the *norA* sequences of CC59 and CC121 strains (Figure 3E). Regarding the allelic products (Figure 3F), we observed an identity ranging between 93.4% (NorA_CC59_ vs. NorAII) and 97.9% (NorA_CC59_ vs. NorAI).

**Figure 4:**
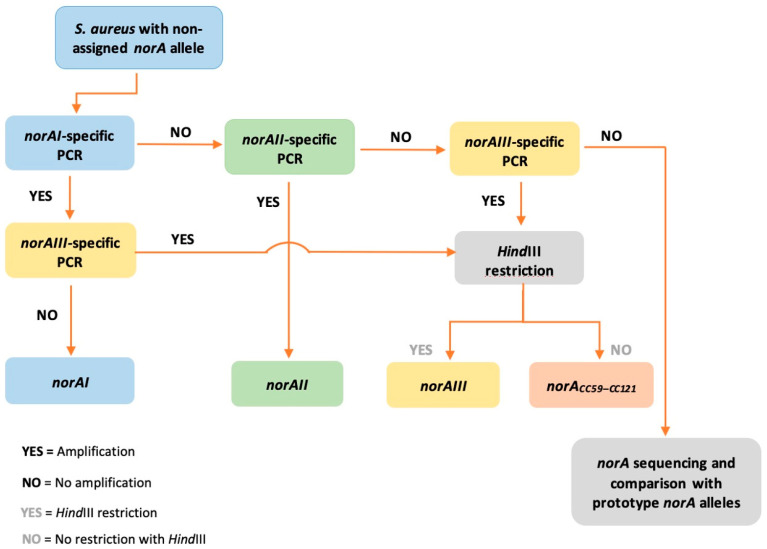
Proposed workflow for the rapid screening of *S. aureus norA* alleles.

Based on our findings, we now delineate a workflow aiming at a rapid screening of *norA* alleles in *S. aureus* (Figure 4). The proposed workflow allowed the detection and differentiation of *norA* alleles in *S. aureus* collections from different settings and hosts, demonstrating the applicability of our PCR-based algorithm to rapidly identify the different *norA* alleles.

### 2.4. Prevalence of S. aureus norA Alleles and Their Association with Specific Clonal Lineages

Applying the proposed workflow to the subset of 52 strains studied, *norAI* was the most prevalent allele, carried by 32 (32/52, 61.6%) strains, followed by *norAII*, present in 17 (17/52, 32.7%) strains. A single strain harbored the *norAIII* allele (1/52, 1.9%). Interestingly, we also detected the recently described *norA_CC59_*–*_CC121_* allele in two strains (2/52, 3.8%) (Table 3). Taking the whole collection in consideration (*n* = 89), assuming isolates from the same clonal lineage harbor the same *norA* allele, the allelic distribution was as follows: *norAI*: 47/89 (52.8%), *norAII*: 39/89 (43.9%), *norA_CC59_*–*_CC121_*: 2/89 (2.2%), and *norAIII*: 1/89 (1.1%).

Analyzing the allelic prevalence according to the host (human vs. companion animal) from which strains were isolated, we observed a predominance of *norAI* for strains of human origin (*n* = 24, 24/34, 70.6%), followed by *norAII* (*n* = 9, 9/34, 26.5%) and *norAIII* (*n* = 1, 1/34, 2.9%). For strains from companion animals, *norAI* and *norAII* were found at somewhat similar frequencies; 23/55 (41.8%) and 30/55 (54.6%), respectively. The *norA_CC59_*–*_CC121_* allele was only identified in two strains (2/55, 3.6%) from one horse and one rabbit. The difference between the prevalence of these alleles among isolates of human or companion animal origin could be explained by the wider dissemination of specific *S. aureus* lineages within those hosts.

In sum, we observed the prevalence of the two alleles *norAI* and *norAII*, which account for up to ~97% of the *norA* variants found in the 89 *S. aureus* isolated from human and companion animals. This result is in accordance with our previous analysis of genomic datasets (from 1038 *S. aureus* strains) [19], which also revealed that *norAI* and *norAII* accounted for over 96% of all genomes screened.

**Figure 5:**
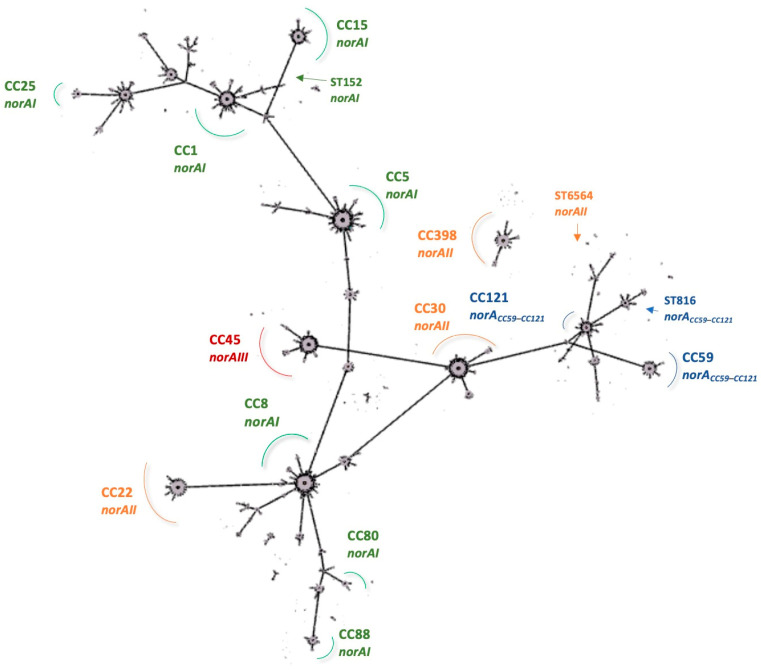
Global distribution of *norA* alleles amongst the main *S. aureus* clonal lineages. The maximum spanning tree was constructed using all *S. aureus* MLST profiles available at PubMLST with the PhyloviZ 2.0 Online software. The clonal lineages differing by up to three loci are linked.

Our previous findings suggested that one could infer which *norA* allele would be expected according to the strain clonal lineage [19]. We now demonstrate this inference to be correct for all strains tested by superimposing the existing information on each strain clonality with the respective *norA* allelic profile (Table 2). The most prevalent allele in this collection, *norAI*, was identified in strains of the clonal complexes CC1, CC5, CC8, and CC15 and also in CC7, CC25, and CC97. We confirmed the presence of *norAII* in CC22, CC30, CC152, CC398, and the newly identified singleton ST6564. As expected, the less frequent alleles *norAIIII* and *norA_CC59_*–*_CC121_* were detected in strains from CC45 and CC121, respectively. We were also able to associate *norA_CC59_*–*_CC121_* with the animal-related lineage ST816 (Table 2). These data allow us to illustrate the global distribution of *norA* alleles among the main *S. aureus* clonal lineages (Figure 5).

### 2.5. Implications for Future Work

The results described in this paper reinforce *norA* as an important element of the *S. aureus* genome as well as of the entire *Staphylococcus* genus. Establishing *norA* as a conserved gene across *Staphylococcus* will assist in better understanding the staphylococcal efflux machinery.

We also demonstrated the presence of several *S. aureus norA* circulating alleles, whose distribution mimics the *S aureus* main strain lineages defined by MLST. We now confirm the applicability of an experimental workflow to detect the different *norA* alleles. The wider application of this workflow by others will obviate the difficulties observed in the recent past years on the detection of the different *norA* alleles and data interpretation, a problem experienced by many researchers that seriously impacted knowledge advance in this area.

The finding that *norA* is part of the *S. aureus* core genome and thus is present in all *S. aureus* strains implies that reporting the detection of this gene is not sufficient to make a direct association with a particular resistance phenotype. To make such a correlation, one must carry out expression analysis of the *norA* gene as well as of other efflux pump genes, as it has been shown that *S. aureus* strains can display different efflux pump gene expression patterns [7,9,34], even under pressure of the same antimicrobial [8,10,12].

The observed *norA* variability may also impact the design of specific inhibitors, an area of growing interest. As pointed out by Brooks and colleagues [35], most of the studies on genes encoding efflux pumps address substrate specificity but fail to take into consideration the conservation of these genes at the strain level. In the case of *norA*, there are two levels of variation to consider: (i) the different circulating alleles and (ii) the intra-allelic nucleotide variation. In particular, most of the studies carried out on the development of NorA inhibitors are based on strain SA-1199 and its derivative, SA-1199B, constructed by Kaatz and colleagues [36]. This strain carries the *norAIII* allele, which is seldom found among isolates of clinical origin. This allele shows 9% differences in the nucleotide sequence compared to the more prevalent *norAI* and *norAII*, that together may account for up to 96% of circulating *S. aureus* lineages.

Further studies should expand this work to other settings to get a global picture of *norA* diversity and distribution. Additional questions to be addressed should include (i) assay of the functionality of the different *norA* alleles; (ii) detailed regulation of gene expression; (iii) design/testing of (specific) efflux inhibitors for each specific allele.

## 3. Materials and Methods

### 3.1. Screening of norA Alleles among Contemporary S. aureus Strains

#### 3.1.1. Bacterial Strains

The *S. aureus* study collection comprised 52 strains causing skin and soft tissue infections (SSTIs) in humans or companion animals. The 25 strains of human origin (9 MRSA and 16 MSSA) were recovered from 26 ambulatory patients over a five-months period in 2014 [30], whereas the 27 strains of animal origin (14 MRSA and 13 MSSA) were recovered from 15 dogs, 6 cats, 4 rabbits, one horse, and one isolate collected from an unknown animal host, all originating from either an academic veterinary laboratory from 2001 to 2018 or a veterinary private diagnostic laboratory during 2017 or 2018 [31]. These 52 *S. aureus* strains are representative of all the PFGE types or sub-types previously detected in a wider collection of 89 *S. aureus* strains (34 from humans and 55 from animals) [30,31] with clonal lineages established by MLST [30,31].

#### 3.1.2. Screening of *norA* Alleles

The primers used for amplification of the different *norA* alleles were the ones proposed earlier [19]. The primers and PCR conditions are discriminated in Table 1. Briefly, the molecular approach proposed is based on the PCR amplification of allele-specific fragments using three pairs of primers, in which the reverse primed is shared. Each PCR reaction was performed with 1.75 mM MgCl_2_, 0.2 mM dNTPs, 0.4 μM of each primer, 1x Taq buffer, and 0.03 U of NZY*Taq* II. All reagents were acquired from NZYTech (Lisbon, Portugal). Amplification products of the *norAIII*/*norA*_CC59_–_CC121_ alleles were further analyzed by restriction of 20 μL of PCR products with 10 U of *Hind*III (New England Biolabs, Ipswitch, MA, USA) at 37 °C for 90 min, followed by gel electrophoresis in 1% agarose gels.

#### 3.1.3. *norA* Sequencing

Representative strains of the major clonal lineages were selected for analysis of the entire *norA* gene. A set of PCRs was previously designed to amplify three regions (A, B, and C) of the *norA* gene, using three pair of primers [19]. PCR products were sequenced at STAB-Vida (Caparica, Portugal), and the sequences were analyzed and assembled with the program SnapGene Viewer v. 5.1.4.1 (GSL Biotech, San Diego, CA, USA; available at snapgene.com). Alignments were made using MEGA v. 7.0.26 [37]. Sequences were aligned against the prototype sequences of the three established *norA* alleles (*norAI*: D90119.1; *norAII*: AB019536.1; *norAIII*: M97169.1) as well as two *norA* sequences related to the *norA*_CC59_–_CC121_ allele (CC59: strain M013, CP003166 [742094:743260]; CC121: strain XQ, CP013137 [180371:181537]).

#### 3.1.4. Relation between *norA* and *S. aureus* Clonal Lineages

The allelic profiles of all *S. aureus* sequence types (ST) described to date (28 October 2022) were retrieved from the PubMLST database (https://pubmlst.org/organisms/staphylococcus-aureus, accessed on 28 October 2022) and used to construct a minimum spanning tree representing the evolutionary relations between clonal lineages using the goeBURST algorithm [38] with the PHYLOViZ 2.0 Online software (https://online.phyloviz.net/index, accessed on 28 October 2022) [39]

### 3.2. Survey of the Gene Coding for the NorA Efflux Pump among the Staphylococcus Genus

Nucleotide sequences of the *norA* gene from 61 different *Staphylococcus* species were obtained from two different sources: reference *norA* genes for the 56 species available at RefSeq [40] and putative *norA* genes obtained from genome projects for the remaining 7 staphylococcal species available in the *Ensembl Bacteria*, release 49 database (https://bacteria.ensembl.org/index.html, accessed on 28 October 2022). This analysis also included the *norA* sequences of the five *Mammalliicoccus* species, all retrieved from the RefSeq repository. Accession numbers for these sequences can be found in Supplementary Material S1.

Sequences were aligned with the *S. aureus norAI* sequence using the Muscle algorithm provided in the MEGA v11 software package [41] and exported for W-IQ-TREE [42] for phylogenetic analysis. After selection of the best substitution model, a maximum likelihood (ML) tree was reconstructed using a GTR+F+I+G4 model, the ultra-fast bootstrapping option [43], and SH-aLRT support values [44] calculated from 1000 replicates. Visualization of the resulting phylogenetic tree was performed with FigTree v1.4.4 (http://tree.bio.ed.ac.uk/software/figtree/, accessed on 20 October 2022).

### 3.3. Characterization of NorA Polypeptide Sequences across Staphylococci

NorA amino acid sequences derived from the 61 sequences previously identified for the evolutionary study of *norA* gene, which include the three *S. aureus norA* prototype alleles and the five *norA* sequences from the *Mammalliicoccus* species (described in detail in Supplementary Material S1), were aligned using the Muscle algorithm included in MEGA v11 [41]. The resulting multiple sequence alignment was exported for W-IQ-TREE [42] for phylogenetic analysis and for Jalview v2.11.2.5 program [45] for conserved motif visualization.

After selection of the best substitution model in W-IQ-TREE [42], a maximum likelihood (ML) tree was reconstructed using an LG+I+G4 model, the ultra-fast bootstrapping option [43], and SH-aLRT support values [44] calculated from 1000 replicates. Visualization of the resulting phylogenetic tree was performed with FigTree v1.4.4 (http://tree.bio.ed.ac.uk/software/figtree/, accessed on 20 October 2022).

In parallel, multiple sequence alignment was visualized and edited with the Jalview v2.11.2.5 program [45]. Transmembrane segments (TMS) and conserved motifs described for MFS transporters with 12 TMS [28] were manually identified and compared between sequences. Consensus and conservation histograms were calculated for each of the TMS and motifs using Jalview.

The in silico platform PHYRE^2^ (Protein Homology/analogY Recognition Engine v2.0) [46] was used to identify the tridimensional structure most similar to the *S. aureus* NorAI (98% identity), namely the c7Lo8z model of NorA protein in complex with Fab36 [28]. Impact of possible residue substitutions on NorA activity was predicted with the SuSPect algorithm [47], based on the c7Lo8z model, producing a table of scores from 0 to 100 according to predicted deleteriousness (0 = neutral to 100 = deleterious). A score of 50 was recommended as a cut-off between neutral and deleterious variants, with extreme scores allowing more confident predictions [47]. In this work, a score of ≥75 was used as cut-off value.

## 4. Conclusions

The results described in this study show that *norA* is part of the staphylococcal genomic patrimony that follows the evolutionary pathway of these bacteria. Our data also suggest an overall conservation of NorA function across staphylococci. The analysis of *norA* variability within several species opens new avenues for the study of the role played by NorA in other staphylococci of clinical relevance. We also described and applied a molecular approach to study *norA* variability within *S. aureus*. This approach confirmed *norA* as a part of the *S. aureus* core genome and rapidly distinguished several circulating alleles. The observed association between *S. aureus* genetic lineages and *norA* alleles is relevant, as it may impact in efflux activity and the design of NorA inhibitors.

## Figures and Tables

**Table 1:** Molecular approach used for differentiation of *norA* alleles.

Allele	Primers (5′ → 3′)	Amplicon Size (nt)	PCR Conditions	Restriction with *Hind*III (nt)
Forward primers				
*norAI*	YonorA (Fw) ^a^: ATATTCAGTTGTTGTCTTAATAT	230	94 °C, 4 min 35 cycles of 94 °C, 30 s; 55 °C, 30 s; 72 °C, 30 s 72 °C, 5 min	No
*norAII*	NorAII (Fw) ^b^: CTGTATTCTTTATATACATCG	391	94 °C, 4 min 35 cycles of 94 °C, 30 s; 57 °C, 30 s; 72 °C, 30 s 72 °C, 5 min	No
*norAIII*	NorAIII (Fw) ^b^: GACCCCTAAAAAAGTTTCGAC	526	94 °C, 3 min 35 cycles of 94 °C, 30 s; 54 °C, 30 s; 72 °C, 30 s 72 °C, 5 min	166 + 360
*norA*_CC59_–_CC121_				No restriction
Reverse primer				
All alleles	NorA2 (Rv) ^a,c^: GCACATCAAATAACGCACCT			

^a^ Sierra et al. [32]; ^b^ Costa et al. [19]; ^c^ all PCRs use NorA2 as the reverse primer.

**Table 2:** Assignment of *norA* allele according to the lineage of the 52 *S. aureus* strains in study.

Clonal Complex (CC) /Clonal Lineages (ST)	Origin (No. of Strains)	Confirmed Allele			
		*norAI*	*norAII*	*norAIII*	*norA_CC59_*–*_CC121_*
Expected allele: *norAI* ^a^					
CC1					
ST188	Companion animal *(n* = 1)	**+**	**-**	**-**	**-**
ST6565	Companion animal *(n* = 1)	**+**	**-**	**-**	**-**
CC5					
ST5	Companion animal (*n* = 3); human (*n* = 2)	**+**	**-**	**-**	**-**
ST105	Companion animal (*n* = 2); human (*n* = 5)	**+**	**-**	**-**	**-**
ST6531	Human (*n* = 1)	**+**	**-**	**-**	**-**
ST6535	Companion animal (*n* = 1)	**+**	**-**	**-**	**-**
CC8					
ST8	Human (*n* = 4)	**+**	**-**	**-**	**-**
ST72	Companion animal (*n* = 2); human (*n* = 1)	**+**	**-**	**-**	**-**
ST6566	Companion animal (*n* = 1)	**+**	**-**	**-**	**-**
CC15					
ST15	Companion animal (*n* = 1); human (*n* = 2)	**+**	**-**	**-**	**-**
Expected allele: *norAII* ^a^					
CC22					
ST22	Companion animal (*n* = 10); human (*n* = 3)	**-**	**+**	**-**	**-**
CC30					
ST30	Human (*n* = 1)	**-**	**+**	**-**	**-**
CC152					
ST152	Human (*n* = 1)	**-**	**+**	**-**	**-**
CC398					
ST398	Companion animal (*n* = 5)	**-**	**+**	**-**	**-**
Expected allele: *norAIII* or *norA*_CC59_–_CC121_^a^					
CC45					
ST278	Human (*n* = 1)	**-**	**-**	**+ ^b^**	**-**
CC121					
ST121	Companion animal (*n* = 1)	**+**	**-**	**-**	**+ ^c^**
No previous association with *norA* allele					
CC7					
ST7	Companion animal (*n* =1); human (*n* = 1)	**+**	**-**	**-**	**-**
CC25					
ST25	Human (*n* = 2)	**+**	**-**	**-**	**-**
CC97					
ST97	Companion animal (*n* =1); human (*n* = 1)	**+**	**-**	**-**	**-**
---					
ST816	Companion animal (*n* = 1)	**+**	**-**	**-**	**+ ^c^**
---					
ST6564	Human (*n* = 1)	**-**	**+**	**-**	**-**

^a^ According to Costa et al. [19]; ^b^ restriction of PCR product with *Hind*III yielded two fragments of ca. 160 and 360 nt, as expected for *norAIII* allele; ^c^ absence of restriction of PCR product with *Hind*III, as expected for *norA_CC59_*–*_CC121_*.

**Table 3:** Prevalence of the different *norA* alleles and correlation with *S. aureus* strain lineages.

*norA* Allele	No. Strains (%)	Host	ST (CC) [30,31]
*norAI*	32 (61.6%)	Human (*n* = 18) Companion animal (*n* = 14)	ST188, ST6565 (CC1); ST5, ST105, ST6531, ST6535 (CC5); ST7 (CC7); ST8, ST72, ST6566 (CC8); ST15 (CC15); ST25 (CC25); ST97 (CC97)
*norAII*	17 (32.7%)	Human (*n* = 6) Companion animal (*n* = 11)	ST22 (CC22); ST30 (CC30); ST152 (CC152); ST398 (CC398); ST6564
*norAIII*	1 (1.9%)	Human (*n* = 1)	ST278 (CC45)
*norA*_CC59_–_CC121_	2 (3.8%)	Companion animal (*n* = 2)	ST121 (CC121); ST816

ST: sequence type; CC: clonal complex.

## Data Availability

All relevant data have been provided in the paper. Raw data can also be provided by the authors upon reasonable request.

## Supplementary Materials

The following supporting information can be downloaded at https://www.mdpi.com/article/10.3390/ijms232315306/s1.

## References

[1] Ubukata K., Itoh-Yamashita N., Konno M. (1989). Cloning and expression of the *norA* gene for fluoroquinolone resistance in *Staphylococcus aureus*. Antimicrob. Agents Chemother..

[2] Yoshida H., Bogaki M., Nakamura S., Ubukata K., Konno M. (1990). Nucleotide sequence and characterization of the *Staphylococcus aureus norA* gene, which confers resistance to quinolones. J. Bacteriol..

[3] Lewis K. (1994). Multidrug resistance pumps in bacteria: Variations on a theme. Trends Biochem. Sci..

[4] Neyfakh A.A., Borsch C.M., Kaatz G.W. (1993). Fluoroquinolone resistance protein NorA of *Staphylococcus aureus* is a multidrug efflux transporter. Antimicrob. Agents Chemother..

[5] Deng X., Ji Q., Liang H., Missiakas D., Lan L., He C. (2012). Expression of multidrug resistance efflux pump gene *norA* is iron responsive in *Staphylococcus aureus*. J. Bacteriol..

[6] Marchi E., Furi L., Arioli S., Morrissey I., Di Lorenzo V., Mora D., Giovannetti L., Oggioni M.R., Viti C. (2015). Novel insight into antimicrobial resistance and sensitivity phenotypes associated to *qac* and *norA* genotypes in *Staphylococcus aureus*. Microbiol. Res..

[7] DeMarco C.E., Cushing L.A., Frempong-Manso E., Seo S.M., Jaravaza T.A.A., Kaatz G.W. (2007). Efflux-related resistance to norfloxacin, dyes, and biocides in bloodstream isolates of *Staphylococcus aureus*. Antimicrob. Agents Chemother..

[8] Huet A.A., Raygada J.L., Mendiratta K., Seo S.M., Kaatz G.W. (2008). Multidrug efflux pump overexpression in *Staphylococcus aureus* after single and multiple in vitro exposures to biocides and dyes. Microbiology.

[9] Kosmidis C., DeMarco C.E., Frempong-Manso E., Seo S.M., Kaatz G.W. (2010). In silico genetic correlations of multidrug efflux pump gene expression in *Staphylococcus aureus*. Int. J. Antimicrob. Agents.

[10] Costa S.S., Falcão C., Viveiros M., Machado D., Martins M., Melo-Cristino J., Amaral L., Couto I. (2011). Exploring the contribution of efflux on the resistance to fluoroquinolones in clinical isolates of *Staphylococcus aureus*. BMC Microbiol..

[11] Furi L., Ciusa M.L., Knight S., Di Lorenzo V., Tocci N., Cirasola D., Aragones L., Coelho J.R., Freitas A.T., Marchi E. (2013). Evaluation of reduced susceptibility to quaternary ammonium compounds and bisbiguanidines in clinical isolates and laboratory-generated mutants of *Staphylococcus aureus*. Antimicrob. Agents Chemother..

[12] Costa S.S., Viveiros M., Rosato A.E., Melo-Cristino J., Couto I. (2015). Impact of efflux in the development of multidrug resistance phenotypes in *Staphylococcus aureus*. BMC Microbiol..

[13] Papkou A., Hedge J., Kapel N., Young B., MacLean R.C. (2020). Efflux pump activity potentiates the evolution of antibiotic resistance across *S. aureus* isolates. Nat. Commun..

[14] Costa S.S., Viveiros M., Pomba C., Couto I. (2018). Active antimicrobial efflux in *Staphylococcus epidermidis*: Building up of resistance to fluoroquinolones and biocides in a major opportunistic pathogen. J. Antimicrob. Chemother..

[15] Marco L., Liliana G., Anna B., Annarita M. (2017). Intrinsic role of coagulase negative staphylococci *norA*-like efflux system in fluoroquinolones resistance. AIMS Microbiol..

[16] Rampacci E., Felicetti T., Pietrella D., Sabatini S., Passamonti F. (2022). Drug efflux transporters in *Staphylococcus pseudintermedius*: In silico prediction and characterization of resistance. J. Antimicrob. Chemother..

[17] Noguchi N., Okada H., Narui K., Sasatsu M. (2004). Comparison of the nucleotide sequence and expression of *norA* genes and microbial susceptibility in 21 strains of *Staphylococcus aureus*. Microb. Drug Resist..

[18] Kaatz G.W., Seo S.M., Ruble C.A. (1993). Efflux-mediated fluoroquinolone resistance in *Staphylococcus aureus*. Antimicrob. Agents Chemother..

[19] Costa S.S., Sobkowiak B., Parreira R., Edgeworth J.D., Viveiros M., Clark T.G., Couto I. (2019). Genetic diversity of *norA*, coding for a main efflux pump of *Staphylococcus aureus*. Front. Genet..

[20] Madhaiyan M., Wirth J.S., Saravanan V.S. (2020). Phylogenomic analyses of the *Staphylococcaceae* family suggest the reclassification of five species within the genus Staphylococcus as heterotypic synonyms, the promotion of five subspecies to novel species, the taxonomic reassignment of five *Staphylococcus* species to *Mammalliicoccus* gen. nov., and the formal assignment of *Nosocomiicoccus* to the family *Staphylococcaceae*. Int. J. Syst. Evol. Microbiol..

[21] Becker K., Schaumburg F., Kearns A., Larsen A.R., Lindsay J.A., Skov R.L., Westh H. (2019). Implications of identifying the recently defined members of the *Staphylococcus aureus* complex *S. argenteus* and *S. schweitzeri*: A position paper of members of the ESCMID Study Group for Staphylococci and Staphylococcal Diseases (ESGS). Clin. Microbiol. Infect..

[22] Schutte A.H.J., Strepis N., Zandijk W.H.A., Bexkens M.L., Bode L.G.M., Klaassen C.H.W. (2021). Characterization of *Staphylococcus roterodami* sp. Nov., a new species within the *Staphylococcus aureus* complex isolated from a human foot infection. Int. J. Syst. Evol. Microbiol..

[23] Chew K.L., Octavia S., Lai D., Lin R.T.P., Teo J.W.P. (2021). *Staphylococcus singaporensis* sp. nov., a new member of the *Staphylococcus aureus* complex, isolated from human clinical specimens. Int. J. Syst. Evol. Microbiol..

[24] Kosecka-Strojek M., Sabat A.J., Akkerboom V., Becker K., van Zanten E., Wisselink G., Miedzobrodzki J., Kooistra-Smid A.M.D.M., Friedrich A.W. (2019). Development and validation of a reference data set for assigning *Staphylococcus* species based on Next-Generation Sequencing of the 16S-23S rRNA region. Front. Cell Infect. Microbiol..

[25] Paulsen I.T., Brown M.H., Skurray R.A. (1996). Proton-dependent multidrug efflux systems. Microbiol. Rev..

[26] Ginn S.L., Brown M.H., Skurray R.A. (2000). The TetA(K) tetracycline/H^+^ antiporter from *Staphylococcus aureus*: Mutagenesis and functional analysis of motif C. J. Bacteriol..

[27] Zhang X.C., Zhao Y., Heng J., Jiang D. (2015). Energy coupling mechanisms of MFS transporters. Protein Sci..

[28] Shang Y., Lv P., Li S., Wang W., Liu Y., Yang C. (2021). Allele-based analysis revealed the critical functions of region 277-297 in the NorA efflux pump of *Staphylococcus aureus*. J. Antimicrob. Chemother..

[29] Brawley D.N., Sauer D.B., Li J., Zheng X., Koide A., Jedhe G.S., Suwatthee T., Song J., Liu Z., Arora P.S. (2022). Structural basis for inhibition of the drug efflux pump NorA from *Staphylococcus aureus*. Nat. Chem. Biol..

[30] Ferreira C., Costa S.S., Serrano M., Oliveira K., Trigueiro G., Pomba C., Couto I. (2021). Clonal lineages, antimicrobial resistance, and PVL carriage of *Staphylococcus aureus* associated to skin and soft-tissue infections from ambulatory patients in Portugal. Antibiotics.

[31] Costa S.S., Ribeiro R., Serrano M., Oliveira K., Ferreira C., Leal M., Pomba C., Couto I. (2022). *Staphylococcus aureus* causing skin and soft tissue infections in companion animals: Antimicrobial resistance profiles and clonal lineages. Antibiotics.

[32] Sierra J.M., Ruiz J., de Anta M.T.J., Vila J. (2000). Prevalence of two different genes encoding NorA in 23 clinical strains of *Staphylococcus aureus*. J. Antimicrob. Chemother..

[33] Schmitz F.J., Hertel B., Hofmann B., Scheuring S., Verhoef J., Fluit A.C., Heinz H.P., Köhrer K., Jones M.E. (1998). Relationship between mutations in the coding and promoter regions of the *norA* genes in 42 unrelated clinical isolates of *Staphylococcus aureus* and the MICs of norfloxacin for these strains. J. Antimicrob. Chemother..

[34] Kosmidis C., Schindler B.D., Jacinto P.L., Patel D., Bains K., Seo S.M., Kaatz G.W. (2012). Expression of multidrug resistance efflux pump genes in clinical and environmental isolates of *Staphylococcus aureus*. Int. J. Antimicrob. Agents.

[35] Brooks L.E., Ul-Hasan S., Chan B.K., and Sistrom M.J. (2018). Quantifying the evolutionary conservation of genes encoding multidrug efflux pumps in the ESKAPE pathogens to identify antimicrobial drug targets. mSystems.

[36] Kaatz G.W., Seo S.M., Ruble C.A. (1991). Mechanisms of fluoroquinolone resistance in *Staphylococcus aureus*. J. Infect. Dis..

[37] Kumar S., Stecher G., Tamura K. (2016). MEGA7: Molecular Evolutionary Genetics Analysis Version 7.0 for Bigger Datasets. Mol. Biol. Evol..

[38] Francisco A.P., Bugalho M., Ramirez M., Carriço J.A. (2009). Global optimal eBURST analysis of multilocus typing data using a graphic matroid approach. BMC Bioinform..

[39] Ribeiro-Gonçalves B., Francisco A.P., Vaz C., Ramirez M., Carriço J.A. (2016). PHYLOViZ Online: Web-based tool for visualization, phylogenetic inference, analysis and sharing of minimum spanning trees. Nucl. Acids Res..

[40] O’Leary N.A., Wright M.W., Brister J.R., Ciufo S., Haddad D., McVeigh R., Rajput B., Robbertse B., Smith-White B., Ako-Adjei D. (2016). Reference sequence (RefSeq) database at NCBI: Current status, taxonomic expansion, and functional annotation. Nucl. Acids Res..

[41] Tamura K., Stecher G., Kumar S. (2021). MEGA11: Molecular Evolutionary Genetics Analysis Version 11. Mol. Biol. Evol..

[42] Trifinopoulos J., Nguyen L.-T., von Haeseler A., Minh B.Q. (2016). W-IQ-TREE: A fast online phylogenetic tool for maximum likelihood analysis. Nucl. Acids Res..

[43] Hoang D.T., Chernomor O., von Haeseler A., Minh B.Q., Vinh L.S. (2018). UFBoot2: Improving the ultrafast bootstrap approximation. Mol. Biol. Evol..

[44] Guindon S., Dufayard S.F., Lefort V., Anisimova M., Hordijk W., Gascuel O. (2010). New Algorithms and Methods to Estimate Maximum-Likelihood Phylogenies: Assessing the Performance of PhyML 3.0. Syst. Biol..

[45] Waterhouse A.M., Procter J.B., Martin D.M.A., Clamp M., Barton G.J. (2009). Jalview version 2: A Multiple Sequence Alignment and Analysis Workbench. Bioinformatics.

[46] Kelley L.A., Mezulis S., Yates C.M., Wass M.N., Sternberg M.J. (2015). The Phyre2 web portal for protein modeling, prediction and analysis. Nat. Protoc..

[47] Yates C.M., Filippis I., Kelley L.A., Sternberg M.J. (2014). SuSPect: Enhanced prediction of single amino acid variant (SAV) phenotype using network features. J. Mol. Biol..

