# Supplementary material for: Occurrence and Variability of the Efflux Pump Gene norA across the Staphylococcus Genus
Source: Int J Mol Sci. 2022 Dec 4;23(23):15306. doi: 10.3390/ijms232315306 (PMC9738427; doi:10.3390/ijms232315306)
Supplement: Supplementary file 1 [file ijms-23-15306-s001.zip › Supplementary Material S2_SSC.pdf]

International Journal of *Molecular Sciences*

MDPI

Supplementary Material S2

Occurrence and variability of the efflux pump gene *norA* across the *Staphylococcus* genus

Carolina Ferreira[1,#], Patrícia Abrantes[1,#], Sofia Santos Costa[1], Miguel Viveiros[1], Isabel Couto[1,*]

[1] Global Health and Tropical Medicine, GHTM, Unit of Medical Microbiology, Instituto de Higiene e Medicina Tropical, IHMT, Universidade NOVA de Lisboa, UNL, 1349-008 Lisbon, Portugal

* Correspondence: icouto@ihmt.unl.pt (IC), Global Health and Tropical Medicine, GHTM, Unit of Medical Microbiology, Instituto de Higiene e Medicina Tropical, IHMT, Universidade Nova de Lisboa, UNL, Rua da Junqueira 100, 1349-008 Lisbon, Portugal. Tel: +351-21-3652652, Fax: +351-21-3632105

#

Table S2. *Staphylococcus aureus* NorA mutational analysis based on SuSPect algorithm available at the PHYRE[2] platform. The putative effect on NorA activity of each possible mutation is given as a score (0→100) and color (blue→red) code, where 0/blue represent no effect and 100/red represents a strong deleterious impact on protein activity.

***S. aureus* NorA**		**Aminoacid substitution**																			
**Position**	**aa**	**A**	**C**	**D**	**E**	**F**	**G**	**H**	**I**	**K**	**L**	**M**	**N**	**P**	**Q**	**R**	**S**	**T**	**V**	**W**	**Y**
**1**	**M**	33	36	50	41	30	37	40	18	43	11	1	40	37	30	41	39	34	17	37	34
**2**	**N**	14	16	8	10	13	23	6	15	2	17	12	1	9	7	6	8	7	19	18	16
**3**	**K**	24	48	50	23	43	51	22	37	1	40	32	20	22	17	4	24	24	45	47	34
**4**	**Q**	9	23	10	6	19	45	11	14	12	18	12	9	8	1	11	8	8	17	21	21
**5**	**I**	16	25	47	39	9	67	30	1	39	1	6	43	20	38	51	23	22	4	31	17
**6**	**F**	21	37	57	53	1	56	37	12	48	9	15	51	29	39	48	29	23	18	6	7
**7**	**V**	10	19	47	42	16	53	41	4	47	7	9	47	12	42	57	21	4	1	31	30
**8**	**L**	36	30	72	70	25	81	65	9	77	1	13	70	41	65	70	57	36	15	42	47
**9**	**Y**	21	29	65	57	6	75	19	21	64	16	14	36	46	49	59	29	35	25	9	1
**10**	**F**	15	30	53	50	1	47	37	9	62	8	13	49	34	52	60	21	24	13	20	15
**11**	**N**	13	16	24	27	27	17	19	19	35	22	18	1	24	27	36	8	10	20	46	33
**12**	**I**	18	23	55	35	23	63	50	1	60	8	8	24	29	35	57	30	17	6	43	33
**13**	**F**	32	38	68	68	1	76	55	33	77	16	23	60	50	69	76	39	48	27	40	26
**14**	**L**	25	29	62	64	17	74	60	8	71	1	9	60	39	55	66	35	26	12	50	45
**15**	**I**	11	17	26	35	13	45	28	1	60	8	12	26	26	47	36	20	16	4	45	23
**16**	**F**	26	30	64	63	1	60	36	24	72	18	15	44	37	56	60	38	30	22	17	11
**17**	**L**	14	19	46	39	10	49	39	6	52	1	5	43	22	34	47	22	11	3	31	30
**18**	**G**	22	52	37	58	56	1	56	58	68	69	54	39	44	60	67	20	46	55	59	65
**19**	**I**	14	29	47	31	10	69	39	1	54	9	8	34	27	21	44	22	19	6	28	15
**20**	**G**	17	41	36	43	22	1	36	48	50	43	27	20	25	34	49	12	18	45	23	30
**21**	**L**	23	26	51	47	14	64	44	4	56	1	4	47	27	41	53	28	26	7	37	22
**22**	**V**	49	56	84	82	64	87	81	13	88	33	39	85	56	66	87	69	50	2	83	63
**23**	**I**	23	46	53	60	19	78	51	1	72	10	14	45	45	59	70	31	29	10	45	33
**24**	**P**	74	91	88	86	91	90	89	87	91	91	87	90	1	88	93	81	76	83	93	91
**25**	**V**	26	57	74	68	40	87	73	8	79	18	37	72	50	65	82	58	39	1	73	48
**26**	**L**	32	43	62	37	19	79	39	13	69	1	7	56	38	49	65	49	25	21	45	46

Table S2 (cont.)

S. aureus NorA		Aminoacid substitution																			
Position	aa	A	C	D	E	F	G	H	I	K	L	M	N	P	Q	R	S	T	V	W	Y
27	P	38	66	62	61	70	71	65	65	72	67	54	65	1	63	76	39	51	61	70	57
28	V	5	12	13	9	6	42	16	2	15	4	6	16	5	13	23	7	5	0	13	8
29	Y	40	58	64	64	8	83	29	17	70	19	23	64	52	43	68	58	43	30	32	0
30	L	13	27	56	32	17	59	45	6	34	1	4	50	27	21	37	19	20	8	38	34
31	K	4	9	7	3	7	20	4	8	0	7	4	3	5	4	2	5	4	7	10	6
32	D	11	21	0	4	23	48	10	14	23	16	16	10	10	8	29	7	14	14	24	15
33	L	49	62	79	63	23	90	73	23	65	1	16	76	47	53	72	59	60	33	51	34
34	G	6	12	8	9	13	0	5	15	9	15	9	4	8	10	11	6	8	12	9	13
35	L	17	35	48	46	19	53	39	9	51	1	15	42	34	30	48	28	25	15	29	24
36	T	6	13	9	10	15	17	10	12	3	10	8	6	7	10	10	4	0	9	16	16
37	G	6	24	14	14	20	1	16	24	20	24	19	8	7	19	20	8	11	20	22	17
38	S	4	11	7	6	6	20	7	10	17	9	7	7	6	6	15	1	4	8	9	14
39	D	10	26	0	4	20	43	9	15	34	17	12	12	11	5	47	9	10	13	14	23
40	L	7	18	40	25	8	52	32	3	44	1	4	22	13	27	41	18	13	4	19	11
41	G	18	47	49	41	44	1	46	53	55	55	43	24	33	32	53	19	27	52	46	53
42	L	11	18	36	31	8	51	16	2	43	0	4	31	11	23	32	20	15	6	6	8
43	L	24	36	63	60	20	82	53	10	68	1	9	57	33	55	64	31	32	11	45	40
44	V	13	28	51	32	14	60	46	5	62	10	8	38	26	34	64	20	15	1	42	25
45	A	1	26	41	39	33	27	41	26	54	26	21	33	17	39	53	6	11	21	44	44
46	A	1	7	30	27	16	28	26	8	37	9	13	28	10	26	38	7	8	2	27	26
47	F	25	38	53	39	0	66	34	35	62	26	19	27	22	45	57	19	30	35	18	4
48	A	1	20	37	33	17	22	33	20	45	18	12	21	8	30	44	6	12	20	34	25
49	L	12	23	47	43	8	52	38	5	55	1	9	45	22	41	50	29	21	9	22	28
50	S	5	12	23	22	16	28	26	16	38	18	9	21	9	24	47	1	5	13	34	35
51	Q	24	51	39	18	41	64	36	37	39	37	24	28	32	1	31	19	28	33	27	30
52	M	12	21	45	43	9	45	40	11	46	5	1	41	22	28	47	19	14	10	35	33
53	I	12	13	41	40	8	59	36	1	51	4	12	38	18	41	49	24	20	3	32	25
54	I	6	6	30	31	4	26	27	0	39	4	4	27	13	29	39	9	6	3	25	18
55	S	13	38	46	33	61	53	50	51	57	48	31	35	41	22	46	1	21	44	66	63
56	P	73	91	87	85	86	94	89	76	90	76	80	89	1	85	92	83	77	83	90	87
57	F	19	35	48	50	0	63	36	7	52	8	14	48	13	37	49	21	23	12	13	6
58	G	8	28	37	42	26	1	32	24	47	26	22	26	30	31	46	15	20	22	11	27
59	G	42	76	70	72	77	0	72	79	78	80	75	57	62	75	75	38	49	76	65	75
60	T	8	26	24	18	21	46	21	18	16	16	14	11	10	20	11	7	1	14	20	18
61	L	27	31	55	53	15	70	49	8	43	1	3	49	29	45	39	40	37	17	15	25
62	A	1	18	45	40	35	27	42	28	54	29	33	41	20	40	54	6	13	14	46	39
63	D	30	42	0	11	46	41	33	45	50	45	42	25	26	28	55	31	34	45	42	46
64	K	7	15	22	11	19	31	7	10	0	11	9	8	13	6	2	7	9	13	17	15
65	L	10	8	26	15	3	41	12	3	17	0	4	16	14	14	16	15	11	4	7	5
66	G	11	31	20	25	32	0	23	36	28	37	29	10	17	28	26	15	23	36	28	29
67	K	13	18	17	11	16	21	10	22	0	21	15	10	8	7	1	8	13	21	19	14
68	K	24	34	37	20	28	35	20	30	0	33	25	19	19	16	4	25	25	20	26	24
69	L	8	10	20	14	6	47	12	3	6	0	3	7	2	8	6	7	7	5	5	8
70	I	24	34	59	62	16	72	56	1	69	6	7	57	16	44	65	30	19	5	50	34
71	I	35	41	65	67	16	81	61	1	74	6	10	65	40	61	72	52	33	12	57	30
72	C	4	0	27	18	7	56	24	3	35	4	5	17	13	18	24	7	8	3	15	14

Table S2 (cont.)

S. aureus NorA		Aminoacid substitution																			
Position	aa	A	C	D	E	F	G	H	I	K	L	M	N	P	Q	R	S	T	V	W	Y
73	I	10	18	47	47	9	62	42	1	57	5	10	44	13	46	37	17	15	4	16	16
74	G	30	37	64	69	66	1	65	70	74	66	61	51	51	68	74	21	42	70	65	69
75	L	38	30	71	66	25	80	63	11	76	1	8	50	42	44	71	41	33	18	56	42
76	I	6	10	29	29	5	33	26	0	39	3	8	28	13	30	37	11	10	1	22	17
77	L	12	12	46	42	10	37	39	4	53	1	7	42	21	38	47	19	17	6	27	26
78	F	40	56	74	60	0	72	63	43	84	27	27	56	61	72	80	40	35	36	29	10
79	S	1	7	14	14	12	20	16	9	25	10	7	9	10	15	33	0	5	13	21	15
80	V	6	8	29	26	7	42	26	1	38	3	8	30	9	26	42	12	9	0	24	13
81	S	6	17	14	25	17	23	29	20	42	22	14	16	18	24	51	1	11	26	38	27
82	E	9	20	4	0	11	37	11	20	24	11	9	7	5	4	30	4	11	12	13	9
83	F	12	20	41	39	0	42	27	7	50	5	10	38	20	40	48	18	16	7	8	8
84	M	6	11	29	26	8	24	24	4	31	1	0	24	9	10	30	11	12	4	15	20
85	F	18	12	53	55	0	58	34	16	64	13	12	52	24	36	63	21	22	22	13	5
86	A	2	44	71	66	49	32	63	45	74	43	45	52	31	63	75	25	47	38	60	44
87	V	8	15	35	23	6	52	30	1	41	4	6	24	11	17	39	17	10	1	14	8
88	G	2	18	22	26	14	1	22	18	29	19	22	11	12	17	21	3	11	13	12	17
89	H	18	35	11	12	26	41	0	31	22	29	30	7	10	9	19	12	12	24	19	11
90	N	13	33	8	16	25	28	3	31	23	25	28	0	17	13	19	5	9	25	38	29
91	F	28	55	58	57	1	74	47	16	53	14	18	62	19	63	70	44	39	19	24	8
92	S	4	9	9	6	8	20	9	8	13	9	9	6	2	9	22	1	4	7	6	9
93	V	17	24	39	22	21	48	32	5	56	6	7	51	17	24	63	21	14	1	18	33
94	L	43	45	67	59	12	83	60	12	74	1	9	66	37	59	57	42	40	20	40	30
95	M	9	17	19	22	5	51	29	1	38	4	0	15	15	19	37	12	13	6	15	8
96	L	2	12	30	18	5	41	23	3	36	0	5	26	11	24	31	11	10	3	13	17
97	S	6	18	28	21	18	30	34	23	47	18	25	20	26	31	51	1	20	23	36	27
98	R	50	63	74	41	62	69	44	52	39	51	47	45	44	36	0	54	43	55	65	44
99	V	5	9	28	24	5	36	24	1	37	3	5	27	10	25	40	10	8	0	22	16
100	I	11	12	31	27	6	38	27	0	40	1	6	30	8	30	38	20	12	3	25	16
101	G	5	21	12	23	21	1	11	25	25	24	18	9	12	8	36	7	11	17	13	24
102	G	20	56	54	44	61	0	53	50	63	65	53	40	40	57	47	37	54	62	39	60
103	M	6	7	26	24	1	35	21	3	27	4	0	22	10	15	26	9	8	3	15	17
104	S	9	12	23	23	12	15	26	25	33	18	14	19	11	16	46	1	9	19	31	35
105	A	1	16	38	31	32	24	29	23	39	21	20	26	22	39	51	9	18	16	30	36
106	G	9	36	45	53	56	1	29	56	59	61	39	27	37	54	61	17	43	48	51	57
107	M	15	35	40	52	15	44	41	14	56	8	1	29	21	36	44	22	24	20	42	29
108	V	23	33	66	48	19	77	62	6	71	19	13	49	39	44	76	31	17	1	58	26
109	M	23	45	59	45	23	71	54	15	63	16	1	42	23	27	52	24	28	18	24	32
110	P	53	83	80	79	87	82	83	74	88	75	78	77	1	73	91	58	54	60	88	88
111	G	6	24	41	45	41	1	40	25	51	36	31	21	13	33	45	11	16	20	37	46
112	V	11	19	59	55	26	64	44	9	69	16	14	57	20	40	74	18	18	1	55	29
113	T	13	25	33	24	16	43	24	17	41	12	8	15	16	18	24	11	1	14	25	22
114	G	3	21	32	36	36	1	31	25	39	38	19	17	15	35	41	8	16	18	31	26
115	L	20	18	56	54	13	80	49	11	62	1	2	54	30	46	58	25	24	14	27	10
116	I	38	73	86	87	55	91	86	2	90	36	37	85	67	86	89	51	65	13	86	73
117	A	2	62	68	67	79	55	73	54	72	68	66	77	51	68	71	41	45	42	83	67
118	D	52	76	1	25	78	84	52	75	76	73	73	47	64	50	77	45	57	71	79	73

Table S2 (cont.)

S. aureus NorA		Aminoacid substitution																			
Position	aa	A	C	D	E	F	G	H	I	K	L	M	N	P	Q	R	S	T	V	W	Y
119	I	33	30	56	52	27	76	38	3	47	11	15	37	41	38	39	27	29	2	51	24
120	S	40	64	44	59	47	73	59	55	67	61	62	54	59	57	74	3	23	52	69	48
121	P	20	39	21	18	43	52	27	41	24	36	32	26	1	22	31	17	16	39	42	39
122	S	1	5	3	2	5	12	3	4	3	3	3	2	2	3	5	0	3	5	7	4
123	H	4	10	4	2	8	20	0	9	2	8	9	4	6	4	6	4	6	10	10	5
124	Q	14	28	3	3	27	52	10	22	8	22	11	8	16	1	8	10	11	23	24	20
125	K	8	20	15	7	16	39	12	15	0	9	7	8	9	7	1	8	11	11	17	16
126	A	1	25	40	35	34	15	37	26	47	23	19	22	7	33	38	8	12	21	42	27
127	K	8	22	26	8	17	33	9	21	0	16	8	8	13	8	3	10	10	21	15	12
128	N	6	25	17	19	20	12	8	14	24	15	13	0	18	12	13	7	9	12	18	11
129	F	45	48	60	60	1	76	47	12	69	13	8	36	41	44	59	37	40	26	36	11
130	G	12	45	46	47	50	1	44	52	54	54	41	29	31	50	54	12	29	49	44	50
131	Y	27	36	53	46	6	70	18	14	35	11	21	28	34	23	34	26	22	17	6	0
132	M	20	34	52	49	12	75	42	7	44	6	1	37	28	25	43	20	28	8	22	17
133	S	11	24	21	16	21	20	23	24	35	23	11	9	18	15	28	1	8	22	32	23
134	A	1	21	41	36	28	32	37	16	48	16	11	39	18	36	50	10	17	14	31	36
135	I	4	9	32	33	9	29	27	1	40	5	5	29	14	31	40	7	10	3	26	19
136	I	23	34	61	61	13	67	38	1	65	12	10	41	38	47	67	26	34	9	39	28
137	N	21	46	31	36	33	32	26	39	45	33	30	1	24	36	32	11	13	33	49	28
138	S	3	6	8	8	4	14	9	4	15	5	2	8	6	8	18	0	2	4	10	12
139	G	15	47	50	54	49	0	49	59	60	60	50	39	32	54	59	18	39	42	50	56
140	F	34	45	57	57	1	59	38	18	58	17	12	44	32	39	58	32	26	24	16	17
141	I	11	26	40	42	21	49	40	1	52	8	7	40	23	43	51	18	16	4	35	27
142	L	9	13	35	33	8	51	29	3	42	1	5	33	16	29	40	18	13	6	23	22
143	G	19	46	43	47	35	0	43	51	52	51	43	33	28	47	52	22	41	50	40	29
144	P	44	62	60	55	59	76	60	52	70	58	37	48	0	48	72	45	56	46	65	48
145	G	2	10	12	13	5	1	10	6	16	5	5	8	3	13	17	5	6	5	9	10
146	I	11	25	45	44	15	62	40	1	54	4	10	44	23	45	53	33	24	5	38	28
147	G	20	33	49	51	48	0	47	50	58	52	49	37	36	52	59	16	39	53	48	54
148	G	14	41	43	43	41	1	40	36	50	29	36	31	17	45	51	19	26	37	35	46
149	F	19	31	43	40	0	58	20	14	53	10	9	41	25	27	43	23	21	11	6	4
150	M	19	35	51	49	13	64	46	6	48	1	1	45	29	34	47	30	23	10	23	24
151	A	1	29	36	31	39	36	30	31	40	32	25	31	18	29	47	13	16	15	38	32
152	E	9	26	4	0	24	31	6	19	13	16	15	13	8	5	19	7	11	15	19	17
153	V	9	18	17	16	2	41	15	3	24	6	9	19	10	24	32	13	9	1	8	6
154	S	9	20	7	10	19	11	11	17	19	24	19	10	16	12	30	1	12	21	15	17
155	H	9	21	10	10	5	22	0	6	19	7	6	5	6	7	11	6	11	14	3	1
156	R	47	77	67	48	69	76	28	67	24	58	62	46	59	35	1	48	43	73	70	47
157	M	1	11	16	16	5	35	14	5	20	4	0	10	5	9	18	5	6	3	5	6
158	P	50	74	68	72	82	85	76	65	83	58	62	78	1	75	87	54	60	54	79	78
159	F	43	45	70	70	0	64	62	36	80	25	34	69	39	73	79	44	63	36	31	22
160	Y	20	34	53	39	4	55	13	21	51	12	16	25	26	28	50	36	32	16	5	0
161	F	11	13	43	41	0	41	29	7	53	9	9	41	26	42	51	12	17	8	13	10
162	A	1	15	46	46	35	44	46	27	59	31	22	31	20	32	61	13	18	19	37	29
163	G	8	27	44	47	29	1	41	37	52	38	40	27	23	46	53	12	23	26	38	46
164	A	0	8	21	20	10	21	21	6	31	6	9	16	4	21	33	6	3	6	21	22

Table S2 (cont.)

S. aureus NorA		Aminoacid substitution																			
Position	aa	A	C	D	E	F	G	H	I	K	L	M	N	P	Q	R	S	T	V	W	Y
165	L	16	19	58	53	15	72	49	7	64	1	10	55	21	39	59	28	26	10	38	36
166	G	12	29	51	56	37	1	49	40	63	42	30	24	24	56	64	16	30	40	49	39
167	I	8	13	39	37	6	44	33	1	47	3	10	35	14	37	46	14	15	1	27	23
168	L	10	17	46	43	10	60	38	4	53	1	6	33	13	39	48	22	15	7	22	28
169	A	1	15	40	45	36	31	30	24	59	22	25	25	16	44	59	11	20	18	32	37
170	F	12	24	52	53	0	56	38	13	58	10	13	46	34	51	63	23	18	14	10	16
171	I	15	14	52	52	14	62	46	1	55	6	14	37	16	51	60	34	22	4	35	23
172	M	10	14	43	38	8	46	35	5	41	3	0	27	12	22	29	19	12	6	16	16
173	S	5	12	23	16	15	30	25	14	35	15	12	17	13	19	37	1	6	13	34	23
174	I	6	10	32	19	6	47	23	2	37	1	5	21	11	27	28	13	10	1	7	7
175	V	6	11	28	23	4	47	17	3	20	1	6	18	8	20	17	10	7	1	10	10
176	L	18	15	31	33	7	63	38	6	32	1	7	43	23	30	30	21	19	9	13	15
177	I	62	70	73	82	44	89	79	2	71	15	30	81	43	68	79	59	58	20	66	64
178	H	11	22	11	6	12	29	0	23	6	14	17	8	5	7	8	9	13	17	18	7
179	D	8	14	0	2	11	26	8	12	11	10	13	3	6	7	15	6	8	12	11	17
180	P	29	52	33	23	56	53	35	39	35	36	32	42	1	37	44	22	24	41	53	42
181	K	8	19	12	6	18	29	7	16	1	12	9	8	8	7	2	10	10	12	13	12
182	K	8	16	15	7	17	27	8	13	1	11	7	7	7	7	3	7	8	11	13	14
183	S	4	6	6	3	8	19	5	7	7	7	6	5	6	6	9	1	5	7	8	9
184	T	5	9	11	7	11	16	12	7	9	9	7	7	7	9	9	7	1	7	12	13
185	T	3	6	7	5	5	17	6	4	6	4	4	5	4	6	5	3	1	4	8	6
186	S	3	6	5	2	6	14	4	5	6	6	4	4	5	4	6	1	4	6	7	8
187	G	3	7	6	5	5	1	5	5	6	4	6	4	4	6	6	4	5	6	6	7
188	F	4	9	9	8	1	17	6	5	10	5	5	9	5	8	7	6	6	6	5	5
189	Q	3	5	3	2	4	14	1	4	4	3	3	4	3	1	4	4	3	3	4	5
190	K	7	11	12	7	10	18	7	9	1	8	8	6	7	6	5	4	7	10	8	7
191	L	9	13	13	10	8	29	14	5	13	1	6	14	8	10	14	9	12	6	9	10
192	E	8	16	5	1	11	26	9	15	11	9	13	10	7	7	16	7	12	10	14	15
193	P	11	22	15	13	17	22	12	16	16	12	17	13	1	15	20	11	14	13	22	16
208	V	2	13	24	25	8	47	25	2	37	4	6	29	11	25	40	17	11	2	17	10
209	I	28	26	68	69	17	65	67	1	65	9	19	58	34	68	65	38	35	9	43	37
210	L	22	22	65	60	14	66	55	7	58	1	9	43	33	42	48	28	27	13	29	27
211	T	16	22	48	35	22	48	42	17	46	22	15	23	19	31	51	11	1	13	32	28
212	L	22	20	63	56	8	62	37	11	66	1	8	52	34	46	63	30	26	16	38	28
213	V	17	19	59	51	16	65	54	5	69	8	11	45	22	38	62	28	19	1	33	30
214	L	21	23	65	59	14	69	38	10	57	1	8	43	39	38	51	26	24	15	28	26
215	S	1	7	14	9	8	22	7	11	22	10	6	6	10	8	21	2	5	10	10	11
216	F	35	50	74	73	1	80	53	24	80	21	24	55	47	58	70	44	42	36	22	19
217	G	18	37	51	50	43	1	61	49	72	51	39	35	31	52	69	26	38	43	40	61
218	L	27	38	62	47	14	65	31	10	68	1	9	38	37	36	48	30	31	16	23	23
219	S	7	23	26	18	17	31	20	22	41	22	11	14	22	13	37	1	11	17	31	18
220	A	1	11	42	29	20	19	32	14	48	16	14	17	11	15	45	10	14	13	38	23
221	F	45	48	74	73	1	81	49	25	81	31	28	60	41	73	67	53	63	36	31	14
222	E	20	40	10	1	20	55	16	23	39	23	20	21	13	9	36	17	21	21	23	20
223	T	15	29	45	30	22	49	40	22	48	24	20	19	15	26	49	12	1	18	34	28
224	L	37	36	79	75	21	79	67	12	76	2	12	54	42	53	71	43	26	14	37	29

Table S2 (cont.)

S. aureus NorA		Aminoacid substitution																			
Position	aa	A	C	D	E	F	G	H	I	K	L	M	N	P	Q	R	S	T	V	W	Y
225	Y	19	33	45	28	2	48	11	12	44	8	13	36	19	31	46	22	19	15	6	1
226	S	8	22	25	24	31	31	32	22	43	24	22	14	8	20	50	1	12	19	30	27
227	L	27	42	64	44	16	84	56	11	70	1	12	59	20	40	57	46	42	17	45	24
228	Y	51	69	77	60	10	76	25	41	80	37	36	70	62	50	59	51	66	41	21	1
229	T	9	17	39	23	15	37	36	13	32	11	12	21	14	23	27	8	1	8	21	22
230	A	1	8	8	6	11	18	7	6	11	7	5	8	5	7	12	1	5	6	10	9
231	D	7	11	1	1	8	27	5	7	13	9	10	6	7	5	17	6	8	10	11	2
232	K	20	42	31	15	34	44	19	31	1	26	21	13	21	12	6	18	20	23	43	23
233	V	4	25	35	32	9	35	18	6	28	9	10	36	19	30	23	29	21	1	31	10
234	N	7	13	6	6	12	5	3	12	7	10	10	1	9	6	7	5	6	10	14	11
235	Y	38	64	73	51	9	60	27	36	66	29	32	60	52	42	64	46	46	37	12	1
236	S	9	20	9	9	26	19	18	25	18	24	19	9	14	14	30	1	3	20	23	28
237	P	24	52	37	32	46	65	47	37	51	33	28	43	1	41	67	33	29	36	43	50
238	K	3	7	7	3	4	16	4	5	0	4	3	3	4	3	1	3	3	5	4	5
239	D	13	28	0	4	20	42	11	18	31	21	17	12	11	7	37	11	14	21	25	33
240	I	28	47	70	68	27	84	62	1	63	16	21	50	48	57	75	42	33	10	41	32
241	S	5	13	22	20	15	19	20	25	24	17	17	13	14	17	41	1	9	25	14	19
242	I	12	25	50	46	9	49	41	1	43	5	8	41	26	46	43	22	19	6	13	16
243	A	1	10	24	31	13	30	24	8	43	10	10	37	9	20	42	10	12	8	18	27
244	I	19	22	56	40	10	76	47	1	49	6	8	49	30	50	60	29	23	6	35	18
245	T	7	15	27	23	11	38	25	10	30	10	7	12	12	17	29	8	1	3	18	15
246	G	6	17	28	23	11	1	27	13	37	16	17	19	12	21	38	9	17	13	13	19
247	G	11	22	28	38	22	1	25	26	45	28	20	11	14	29	35	12	20	25	26	23
248	G	15	48	53	43	49	1	42	55	60	46	33	29	33	43	58	19	30	39	44	50
249	I	12	16	44	43	11	55	39	1	54	6	9	39	16	40	49	19	25	2	22	26
250	F	3	11	32	22	0	31	21	7	30	7	8	20	12	21	39	13	14	8	9	7
251	G	13	41	32	48	34	1	30	38	55	37	29	22	34	38	37	17	29	36	37	39
252	A	1	18	40	36	19	42	36	12	50	15	15	28	9	28	48	12	15	10	33	35
253	L	17	19	46	41	9	59	36	6	51	1	6	39	19	24	34	29	18	3	29	26
254	F	15	19	47	44	1	48	31	10	55	10	10	29	18	46	54	20	21	10	15	11
255	Q	38	67	60	39	53	68	57	56	66	51	33	44	39	2	53	34	42	55	51	62
256	I	8	17	32	30	9	30	27	1	38	4	5	16	8	20	20	12	11	1	17	17
257	Y	13	18	37	25	1	33	7	7	21	6	8	16	9	14	15	13	9	8	3	0
258	F	17	31	57	51	1	54	31	11	63	10	17	51	33	43	46	32	23	16	15	12
259	F	17	33	51	49	1	39	25	9	59	12	13	35	23	49	45	21	18	13	17	8
260	D	9	17	0	4	13	17	12	17	20	15	15	7	6	12	17	6	11	14	20	15
261	K	21	41	44	21	27	48	26	23	1	23	14	18	12	14	5	21	19	25	14	19
262	F	17	37	53	51	1	50	38	8	46	6	13	39	35	38	44	26	28	13	10	9
263	M	9	17	20	15	9	39	15	6	21	5	1	19	11	12	18	8	10	5	19	16
264	K	8	13	10	6	9	22	10	10	1	11	6	5	7	8	3	8	8	16	12	12
265	Y	17	33	29	31	6	56	5	16	12	13	15	17	22	18	8	17	15	18	5	0
266	F	17	21	32	23	1	31	15	9	22	8	4	18	19	31	20	17	18	13	9	5
267	S	6	13	8	12	16	9	8	12	10	15	7	6	8	8	12	1	3	14	12	18
268	E	19	39	11	1	36	47	15	27	21	24	21	20	10	13	26	17	24	25	36	35
269	L	9	11	22	13	7	31	13	4	12	1	3	16	7	11	13	10	9	5	9	8
270	T	9	19	25	25	17	30	15	10	13	12	9	10	7	15	10	9	1	10	13	17

Table S2 (cont.)

S. aureus NorA		Aminoacid substitution																			
Position	aa	A	C	D	E	F	G	H	I	K	L	M	N	P	Q	R	S	T	V	W	Y
271	F	26	42	60	63	1	75	49	13	61	11	12	59	28	41	53	28	21	13	15	12
272	I	38	54	79	78	28	74	71	1	83	13	16	74	55	73	67	52	36	11	74	55
273	A	1	7	24	19	10	18	20	2	19	7	8	13	7	10	14	5	8	5	14	13
274	W	13	16	51	34	7	39	33	12	46	10	14	34	19	30	39	23	18	14	0	9
275	S	8	9	27	28	19	19	32	21	45	17	21	14	23	29	47	1	10	18	38	28
276	L	27	26	68	67	15	67	46	11	75	1	10	50	41	46	69	37	34	19	47	34
277	L	8	9	34	28	6	43	26	1	40	1	5	28	9	26	36	12	11	4	15	18
278	Y	6	6	29	21	1	25	6	4	27	3	4	15	15	16	15	10	6	4	4	0
279	S	5	13	17	25	16	25	28	18	28	18	9	13	21	18	38	1	9	20	35	21
280	V	1	7	22	27	7	19	25	3	39	5	5	19	10	19	39	11	7	1	21	19
281	V	6	8	30	23	9	32	25	1	38	4	6	26	7	26	30	13	9	2	21	13
282	V	6	11	41	24	9	25	40	4	55	6	7	39	12	25	60	12	12	1	32	19
283	L	22	38	70	65	15	69	63	11	77	1	9	51	39	58	73	38	28	18	33	27
284	I	6	12	33	30	4	11	26	2	36	3	6	28	8	29	34	13	10	1	19	10
285	L	16	21	55	54	15	62	46	8	67	1	7	54	19	50	57	30	27	11	19	25
286	L	32	21	71	67	18	71	59	10	72	1	9	65	29	40	67	31	22	16	35	29
287	V	4	10	21	25	8	29	24	1	36	4	5	17	5	25	44	9	6	1	13	9
288	F	15	26	29	38	2	48	19	3	46	4	13	26	13	19	33	29	18	13	10	8
289	A	1	14	28	37	22	30	22	19	32	20	13	28	15	26	46	4	13	11	36	36
290	N	5	11	1	6	7	11	1	7	5	7	5	1	5	4	5	3	3	6	8	7
291	G	6	23	12	14	17	1	11	19	22	18	19	5	11	11	14	2	8	23	16	15
292	Y	22	43	43	33	4	55	11	15	36	15	27	23	24	24	36	28	24	19	6	0
293	W	32	61	71	53	25	60	62	35	76	27	46	70	33	52	75	46	29	39	0	24
294	S	2	6	8	4	5	15	4	4	10	4	2	6	3	4	14	1	1	4	4	6
295	I	19	31	45	60	11	62	54	1	71	6	8	57	25	41	56	30	28	5	35	21
296	M	11	25	43	45	8	43	27	6	51	4	1	44	17	34	52	21	18	7	17	15
297	L	6	10	35	32	3	42	26	3	41	2	5	34	8	23	41	16	13	1	11	19
298	I	11	14	52	53	13	48	48	1	64	6	13	49	16	52	58	17	16	4	37	19
299	S	5	11	24	21	14	28	22	14	37	11	7	13	12	14	32	1	6	11	28	18
300	F	26	24	69	68	1	58	53	18	77	18	21	65	34	66	76	32	30	20	31	18
301	V	2	9	37	31	6	45	22	1	46	4	6	26	8	30	49	17	13	2	16	20
302	V	12	22	50	39	8	46	40	3	57	7	7	33	16	23	61	22	16	1	17	14
303	F	45	56	84	69	1	57	75	50	89	44	38	77	74	81	87	43	49	49	37	21
304	I	14	16	57	34	12	54	49	1	65	5	8	40	22	36	63	21	15	5	30	23
305	G	14	41	62	50	29	1	54	47	67	43	48	48	42	63	59	25	50	57	56	62
306	F	36	51	55	47	1	61	39	19	61	25	22	35	51	49	74	34	34	27	24	15
307	D	15	31	1	11	31	29	28	23	53	28	18	12	16	17	61	9	16	24	24	43
308	M	30	40	71	65	21	58	60	14	67	4	1	57	35	48	60	34	34	19	38	46
309	I	20	26	49	61	14	60	54	1	70	7	12	40	37	45	67	31	21	6	42	31
310	R	30	53	59	34	33	61	27	38	29	31	20	25	30	23	2	35	28	43	50	32
311	P	67	89	89	86	91	92	89	82	93	83	85	83	1	86	94	73	69	80	91	91
312	A	1	17	62	54	33	45	55	28	67	27	28	35	17	51	69	15	19	23	60	54
313	I	16	27	52	49	13	56	42	1	60	2	9	27	26	27	44	20	18	6	41	28
314	T	24	45	41	44	38	64	56	32	56	32	22	19	20	24	50	14	1	26	53	42
315	N	11	42	18	30	45	35	21	30	41	28	33	1	30	27	31	8	8	28	52	31
316	Y	34	42	72	51	12	64	17	25	68	18	19	60	55	33	57	43	35	25	14	1

Table S2 (cont.)

S. aureus NorA		Aminoacid substitution																			
Position	aa	A	C	D	E	F	G	H	I	K	L	M	N	P	Q	R	S	T	V	W	Y
317	F	11	27	45	33	0	63	28	6	55	5	5	32	26	26	55	16	18	8	21	2
318	S	25	51	51	40	63	61	52	57	68	59	31	45	49	49	60	2	23	45	64	66
319	N	11	20	9	10	22	24	8	21	9	15	8	1	17	10	9	9	11	16	24	14
320	I	21	28	56	32	20	57	26	1	46	8	11	38	38	38	40	31	23	8	51	36
321	A	2	43	38	43	57	34	42	45	51	48	54	62	25	57	55	17	32	31	66	45
322	G	4	13	6	6	13	0	6	10	5	11	11	4	4	7	7	5	6	10	9	12
323	E	7	15	1	2	15	23	6	15	8	15	13	2	6	5	11	7	7	10	14	13
324	R	23	46	41	21	36	42	15	34	10	27	21	15	39	12	1	28	22	47	39	28
325	Q	31	60	49	26	62	65	31	46	32	56	26	49	37	1	26	30	39	43	58	38
326	G	46	81	77	81	83	1	77	85	84	84	80	54	61	83	83	53	61	84	79	82
327	F	10	28	41	21	0	46	25	9	31	7	8	37	26	23	24	13	12	12	10	5
328	A	1	44	64	56	38	46	48	24	72	27	25	49	36	46	72	27	30	17	57	32
329	G	6	33	34	38	17	1	32	22	43	17	9	10	23	17	42	6	14	29	29	21
330	G	26	69	73	66	66	1	72	78	80	77	73	49	56	76	77	29	53	76	70	73
331	L	28	31	69	64	20	70	59	12	71	1	10	64	42	61	66	36	32	15	31	31
332	N	31	51	44	46	30	57	28	36	47	33	27	1	48	29	50	23	25	38	54	26
333	S	13	39	39	26	29	36	26	43	53	43	18	11	32	20	49	1	17	37	52	32
334	T	10	29	47	43	20	46	44	27	53	20	13	33	25	42	51	8	1	20	38	28
335	F	18	24	59	58	1	59	43	14	67	13	15	57	32	56	67	24	27	21	21	10
336	T	11	24	22	22	17	36	20	13	27	11	8	11	17	12	16	10	1	12	20	25
337	S	16	34	21	34	24	46	24	42	53	41	20	16	28	22	47	1	19	34	41	26
338	M	11	22	43	38	9	42	36	6	44	3	1	37	20	30	43	24	16	6	20	29
339	G	18	44	63	65	60	1	60	51	70	54	43	47	46	67	71	29	39	49	58	65
340	N	16	35	20	18	21	35	14	20	26	25	13	1	27	16	19	11	16	24	36	23
341	F	34	60	74	73	2	65	64	20	80	37	25	72	56	73	79	43	42	31	53	41
342	I	8	13	41	39	7	39	36	1	48	5	9	38	15	39	47	19	16	2	32	27
343	G	27	66	72	62	69	1	69	73	78	76	65	49	45	75	77	37	50	72	65	73
344	P	54	81	82	79	78	79	82	80	87	82	75	82	2	80	89	60	68	70	84	85
345	L	13	28	53	47	11	66	43	6	57	1	6	48	12	43	55	26	16	10	17	19
346	I	11	17	44	44	11	65	40	1	55	4	9	43	24	42	53	21	18	2	23	23
347	A	1	17	49	29	19	15	44	31	46	21	15	48	23	45	60	10	17	19	37	39
348	G	25	57	63	66	51	1	62	49	73	55	43	36	54	64	74	31	35	64	53	62
349	A	1	16	27	27	9	37	23	9	33	9	9	19	9	17	25	11	12	8	11	11
350	L	25	41	72	66	22	74	60	10	75	1	9	65	33	65	72	40	38	15	46	49
351	F	30	55	68	65	1	75	50	22	74	20	19	64	53	52	59	37	51	29	33	8
352	D	24	48	1	9	49	54	25	42	57	44	36	20	31	21	53	18	23	40	50	43
353	V	10	20	38	28	12	45	20	3	24	6	7	24	14	20	27	18	10	1	13	10
354	H	14	26	11	15	9	27	0	25	20	18	21	3	17	12	18	10	14	19	16	6
355	I	11	16	22	32	11	25	15	1	38	6	6	19	11	22	27	13	11	2	25	10
356	E	14	39	6	1	21	32	9	23	18	19	27	9	12	6	16	10	16	27	21	16
357	A	1	12	15	13	4	19	12	9	18	8	9	20	8	17	24	6	9	14	10	7
358	P	45	72	70	66	67	72	69	70	76	71	60	71	1	68	79	51	51	56	61	75
359	I	9	16	35	34	4	49	29	1	40	1	5	30	9	31	38	20	15	3	13	6
360	Y	32	51	70	60	7	52	21	21	70	20	20	59	49	56	64	42	34	28	8	1
361	M	24	38	65	57	15	71	52	11	63	8	1	56	29	44	59	31	27	11	24	25
362	A	1	16	58	50	30	26	51	19	65	27	32	48	25	51	62	14	23	20	31	48

Table S2 (cont.)

S. aureus NorA		Aminoacid substitution																			
**Position**	**aa**	**A**	**C**	**D**	**E**	**F**	**G**	**H**	**I**	**K**	**L**	**M**	**N**	**P**	**Q**	**R**	**S**	**T**	**V**	**W**	**Y**
**363**	**I**	12	14	57	56	17	38	51	1	65	8	15	48	32	55	65	20	19	5	46	22
**364**	**G**	8	25	40	38	17	1	34	7	46	18	24	25	16	42	47	21	20	17	26	39
**365**	**V**	16	30	64	58	19	63	59	5	74	10	13	65	24	60	75	37	24	1	53	42
**366**	**S**	4	7	17	15	8	28	18	8	29	7	2	14	11	13	35	1	6	10	21	15
**367**	**L**	21	35	70	60	16	70	57	8	74	1	10	63	44	60	69	49	34	15	46	42
**368**	**A**	1	6	28	23	3	19	23	5	34	6	7	18	8	22	36	7	7	5	20	20
**369**	**G**	19	30	62	65	58	1	61	44	72	42	50	47	50	66	72	26	41	40	30	64
**370**	**V**	7	10	39	31	7	52	34	1	32	4	6	35	15	34	52	13	10	1	11	20
**371**	**V**	15	18	61	53	16	48	53	5	68	12	9	59	28	55	63	33	20	1	27	30
**372**	**I**	22	22	69	66	17	77	60	1	76	7	14	65	31	66	73	50	26	7	46	34
**373**	**V**	6	7	38	30	8	36	32	1	46	4	4	35	15	31	50	10	8	1	22	18
**374**	**L**	16	20	52	45	3	74	37	6	39	1	5	46	25	32	37	24	19	9	9	12
**375**	**I**	11	18	44	40	7	39	20	1	46	4	5	37	22	24	23	18	12	2	27	12
**376**	**E**	8	25	7	1	13	30	8	15	14	12	11	12	10	4	13	7	10	9	8	16
**377**	**K**	12	28	35	11	18	40	17	20	1	25	11	17	14	11	5	20	12	27	15	22
**378**	**Q**	2	9	8	2	10	35	3	10	4	5	5	4	6	0	4	5	6	9	9	8
**379**	**H**	12	19	17	7	15	44	0	19	10	4	14	9	9	8	7	9	10	20	17	6
**380**	**R**	23	28	40	15	26	46	19	20	7	20	18	11	19	6	1	23	17	32	26	24
**381**	**A**	1	15	25	17	22	23	21	18	14	12	9	15	14	13	15	8	9	13	23	21
**382**	**K**	18	26	28	9	25	36	12	25	1	25	17	10	18	9	5	12	18	27	24	20
**383**	**L**	14	13	28	21	10	32	18	5	26	1	4	23	20	22	25	22	16	6	14	14
